# Endoscopic submucosal dissection for an atypical small verrucous carcinoma: a case report

**DOI:** 10.1186/s13256-016-0866-y

**Published:** 2016-03-25

**Authors:** Takahiro Abe, Masayuki Kato, Munenori Itagaki, Sigeharu Hamatani, Yosuke Kawahara, Shuji Ito, Yoshio Aizawa, Koji Matsuda, Kazuki Sumiyama

**Affiliations:** 1 Department of Endoscopy, The Jikei University Katsushika Medical Center, 6-41-2 Aoto Katsushika-ku, Tokyo, 125-8506 Japan; 2 Department of Gastroenterology and Hepatology, The Jikei University Katsushika Medical Center, 6-41-2 Aoto Katsushika-ku, Tokyo, 125-8506 Japan; 3 Department of Pathology, Clinical Service, The Jikei University Katsushika Medical Center, 6-41-2 Aoto Katsushika-ku, Tokyo, 125-8506 Japan; 4 Department of Endoscopy, The Jikei University School of Medicine, 3-25-8 Nishishinbashi Minato-ku, Tokyo, 105-8461 Japan

**Keywords:** Verrucous carcinoma, Esophagus, Endoscopic submucosal dissection

## Abstract

**Background:**

Esophageal verrucous carcinoma is a rare variant of esophageal squamous cell carcinoma. In most cases, verrucous carcinoma presents as an exophytic, slow-growing mass with an extensive superficial growth pattern. Symptoms often include an insidious onset of dysphagia resulting in weight loss. In a patient presenting with super early-stage verrucous carcinoma, we were able to eliminate the aberration using endoscopic submucosal dissection.

**Case presentation:**

An asymptomatic 68-year-old Asian man was found to have an abnormality in his esophagus. The abnormality was discovered, by chance, in a barium study for a health checkup. Esophagogastroduodenoscopy revealed a 1-centimeter polypoid lesion covered with squamous epithelium. Biopsies showed squamous high-grade intraepithelial neoplasia. An endoscopic submucosal dissection was performed and the histopathological findings showed a well-differentiated squamous cell carcinoma with hyperkeratosis with a church spire configuration. These features are consistent with the growth pattern of verrucous carcinoma.

**Conclusions:**

Verrucous carcinoma can manifest as a small mass with nonclinical symptoms and endoscopic submucosal dissection is useful as a curative treatment. We must consider that verrucous carcinoma can manifest as appearance of a polyp that is not papillary or warty-like with and without extensive superficial growth appearance.

## Background

Esophageal verrucous carcinoma (VC) is a rare variant of esophageal squamous cell carcinoma. In most cases, it usually presents as an exophytic, slow-growing mass emerging as papillary or warty-like, with an extensive superficial growth pattern [1–6]. Diagnosis is often delayed resulting in surgical treatment at an early stage of VC [2–6]. Endoscopic submucosal dissection (ESD) is an advanced endoscopic technique that allows for the complete en bloc resection and possible cure of superficial gastrointestinal neoplasms [7–9]. We here report that VC was identified in a small atypical VC and was completely cured by an ESD.

## Case presentation

**Fig. 1:**
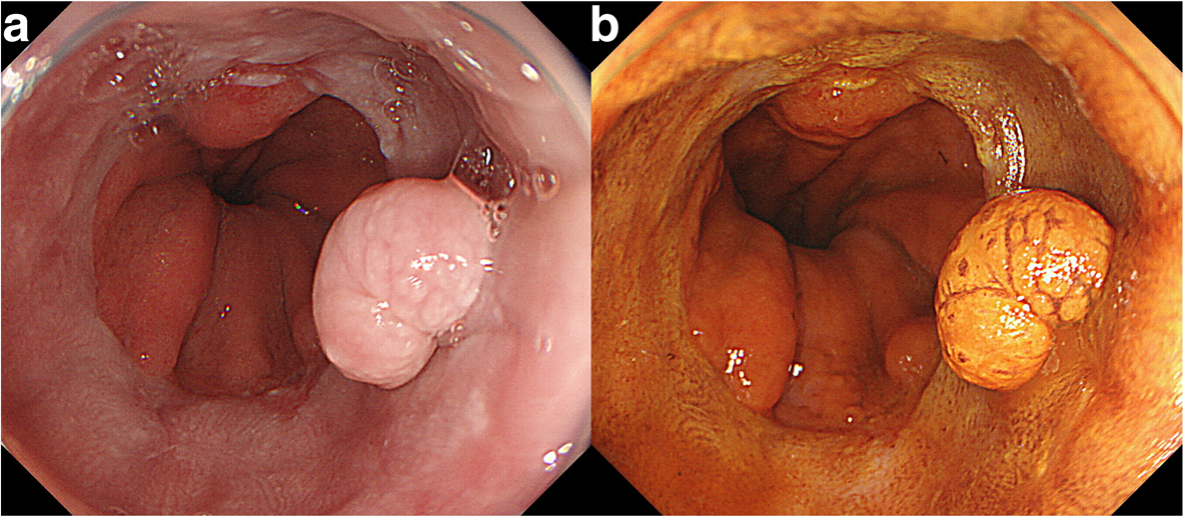
**a** An endoscopic picture showing a polypoid lesion in the distal esophagus. **b** Endoscopic image stained by iodine. The lesion is within the iodine-stained area

**Fig. 2:**
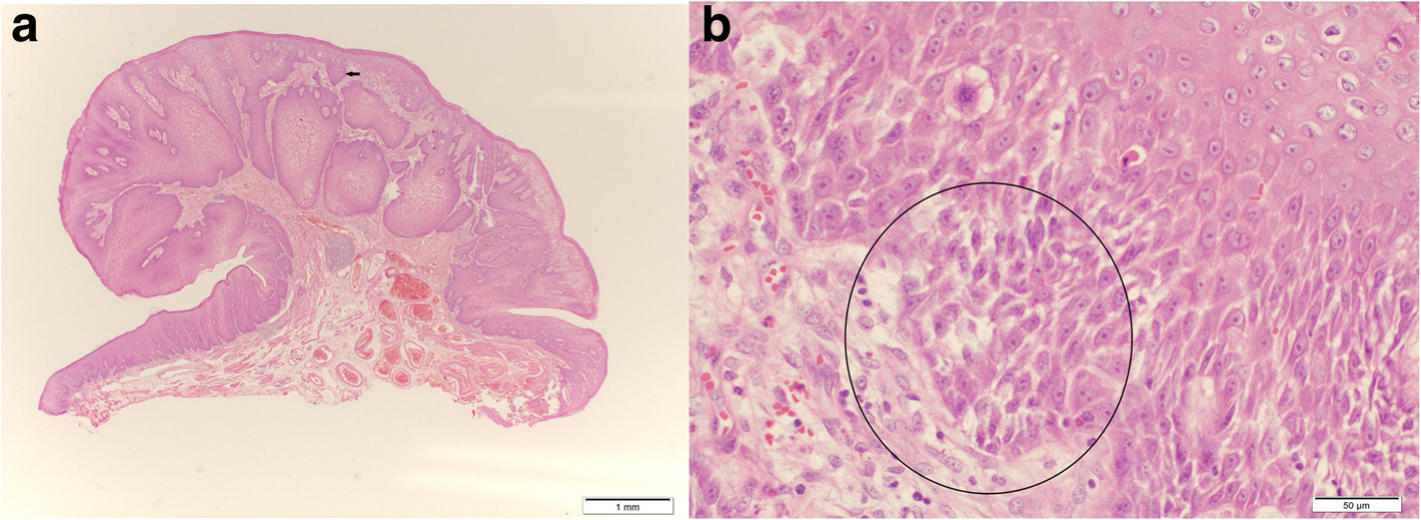
**a** Photomicrograph of the resected esophageal specimen shows epithelial downgrowth and invasive findings (H&E, original magnification, ×20). **b** High-magnification image shows well-differentiated squamous cell carcinoma and focal hyperkeratosis with a church spire configuration (H&E, original magnification, ×400). *Black arrow* and *circle* indicate a focal hyperkeratosis with a church spire configuration

By chance, an asymptomatic 68-year-old Asian man was found to have an abnormality in his esophagus by barium study for mass screening. He was referred to our hospital and underwent an esophagogastroduodenoscopy which showed a 1-centimeter polypoid appearance covered with squamous epithelium in the distal esophagus (Fig. 1). Biopsies showed squamous high-grade intraepithelial neoplasia. An endoscopic submucosal dissection (ESD) was performed and the histopathological findings showed a well-differentiated squamous cell carcinoma with hyperkeratosis with a church spire configuration (Fig. 2). These features were consistent with the growth pattern of VC. No vessel invasion or lymphatic permeation was recognized of this resected specimen.

## Discussion

Esophageal verrucous carcinoma (VC) is a rare variant of esophageal squamous cell carcinoma. The majority of the cases are associated with smoking, reflux esophagitis, alcohol use, human papilloma virus (HPV), achalasia, and other chronic inflammatory conditions. An incidence rate of such cancer has been shown to be higher in males than females, with a ratio of 2:1, and seen in the age group from 35 to 80 years [1].

We ascertained two important clinical issues. VC can manifest as a small mass without clinical symptoms and ESD is useful as a curative treatment.

With regard to the first clinical issue, VC can manifest as a small mass without clinical symptoms. VC of the esophagus is a rare malignant epithelial tumor representing a variant of squamous cell carcinoma. VC was first reported in 1967 [10]. Since then, 44 cases have been reported in the literature. The endoscopic appearance of VC usually presents with an exophytic, whitish, wart-like mucosal surface, and is typically diagnosed months to years after the onset of symptoms such as dysphagia and weight loss [1–6]. Most esophagus squamous cell carcinomas are not stained with a Lugol iodine due to the fact they contain lesser amounts of glycogen. VC, however, has an increased amount of glycogen due to a slow growth pattern. A PubMed MEDLINE search was performed at the Jikei University Library of Medline using the keywords “verrucous cancer” and “esophagus”. Forty-four case reports were determined before October 19, 2015. In forty-three of the 44 cases (or 97.7 %) VC was found to have the appearance of an exophytic, whitish and wart-like mucosal surface, except for one report [11]. One case reported VC with an atypical expression. Tajiri *et al*. reported the endoscopic appearance as an elevated papillary lesion, which was quite different from our findings. We therefore assume that the incipience of VC has various endoscopic forms.

Concerning the second clinical issue, we learned that ESD is useful for curative treatment [10–12]. In the last decade, ESD, a therapeutic endoscopic technique, has been used to excise superficial GI neoplastic lesions. ESD is an alternative, or replacement therapy, to endoscopic mucosal resection (EMR). An ESD is performed using the following technique:

1. Circumferential markings are made a few millimeters outside and around the margin of the lesion using brief bursts of cautery with a Dual knife (KD-650L; Olympus Medical Systems, Tokyo, Japan).
2. Next, submucosal injections are performed along the perimeter of the markings to raise the lesion. A solution of 0.5 % sodium hyaluronate (Mucoup; Seikagaku Corporation, Tokyo, Japan) in 10 % glycerine solution with 0.025 % epinephrine and 0.05 % indigo carmine is used.
3. Third, an incision is made along the perimeter of the markings. This isolates the injected area.
4. Lastly, using an endoscopic knife, the raised superficial lesion is filleted away from the submucosa and removed.

The case described above is the first known report using ESD as a treatment for early-stage VC. Table 1 (below) shows the methods of treatment for VC since it was first reported [10]. Twenty-six cases were surgically treated and only one by EMR [11]. Because of the slow-growing tumor character causing late-stage detection, most cases are not candidates for noninvasive curative treatments like EMR or ESD. Because we identified the tumor in our patient to be VC in an early stage, we were able to use ESD as a treatment. We reason that ESD will decrease the local recurrence rate after endoscopic tissue resection [12–14]. Our patient was thereby cured “en bloc” using a minimally invasive dissection technique instead of surgical resection.

**Table 1:** Forty-four reports of the treatment methods of verrucous carcinoma

Treatment	
Operation	23
Radiation	2
Chemotherapy	1
Chemotherapy/radiation	4
Chemotherapy/radiation/operation	3
Endoscopic mucosal resection	1
Medication	1
Conservative	7
Unknown	2

## Conclusions

VC can manifest as a small mass without clinical symptoms. ESD is a useful curative treatment in the removal of VC. It is further understood that VC can manifest with an atypical polyp appearance and not with its typical papillary or warty-like appearance. It is noteworthy to consider that unknown expressions of VC exist, causing future undetected VC cases. ESD can be a curative treatment if VC lesions are detected early with superficial growth. Subsequent reports should be collected and analyzed to determine whether “hidden” VC cases might be detected and diagnosed and whether ESD use may contribute to curative treatment.

## Consent

Written informed consent was obtained from the patient for publication of this case report and any accompanying images. A copy of the written consent is available for review by the Editor-in-Chief of this journal.

## Abbreviations

EMR: endoscopic mucosal resection
ESD: endoscopic submucosal dissection
VC: verrucous carcinoma
